# The gut microbiome of freshwater Unionidae mussels is determined by host species and is selectively retained from filtered seston

**DOI:** 10.1371/journal.pone.0224796

**Published:** 2019-11-13

**Authors:** Eric A. Weingarten, Carla L. Atkinson, Colin R. Jackson

**Affiliations:** 1 Department of Biology, University of Mississippi, University, Mississippi, United States of America; 2 Department of Biological Sciences, University of Alabama, Tuscaloosa, Alabama, United States of America; Universidade do Porto, PORTUGAL

## Abstract

Freshwater mussels are a species-rich group of aquatic invertebrates that are among the most endangered groups of fauna worldwide. As filter-feeders that are constantly exposed to new microbial inoculants, mussels represent an ideal system to investigate the effects of species or the environment on gut microbiome composition. In this study, we examined if host species or site exerts a greater influence on microbiome composition. Individuals of four co-occurring freshwater mussel species, *Cyclonaias asperata*, *Fusconaia cerina*, *Lampsilis ornata*, and *Obovaria unicolor* were collected from six sites along a 50 km stretch of the Sipsey River in Alabama, USA. High throughput 16S rRNA gene sequencing revealed that mussel gut bacterial microbiota were distinct from bacteria on seston suspended in the water column, and that the composition of the gut microbiota was influenced by both host species and site. Despite species and environmental variation, the most frequently detected sequences within the mussel microbiota were identified as members of the Clostridiales. Sequences identified as the nitrogen-fixing taxon *Methylocystis sp*. were also abundant in all mussel species, and sequences of both bacterial taxa were more abundant in mussels than in water. Site physicochemical conditions explained almost 45% of variation in seston bacterial communities but less than 8% of variation in the mussel bacterial microbiome. Together, these findings suggest selective retention of bacterial taxa by the freshwater mussel host, and that both species and the environment are important in determining mussel gut microbiome composition.

## Introduction

North America is home to the greatest diversity of freshwater mussels in the world, with mussel biodiversity principally concentrated in riverine systems of the Southeastern United States [1,2]. Freshwater mussels (families Margaritiferidae and Unionidae) were once the dominant invertebrates in eastern U.S. streams [3] but are now the most imperiled organisms in North America [4,5]. This shift in mussel biodiversity is attributed to the combined effects of invasive species competition, human alterations to hydrology, and dissolved contaminants [6]. Mussels play an essential role in aquatic ecosystem function by coupling the pelagic and benthic compartments of streams through their filter-feeding activity [7], which can stimulate primary production [8] and alleviate nutrient limitation [9,10]. While freshwater mussels are important for their ecological function and from a conservation and biodiversity standpoint, little is known of their associated microbiome, even though as filter feeders their gut microbiome may be particularly sensitive to environmental variation.

The factors that drive the assembly of microbial communities have been explored for many organisms and environments [11–13], but rarely so for freshwater bivalves. Most of the current literature describing bivalve microbiota are from marine species. For marine bivalves, several studies have focused on the Eastern Oyster, *Crassostrea virginica*, but even patterns for this species are unclear [14–18]. Gut communities of *C*. *virginica* differed by site, individual, and even between compartments within an individual in samples collected from coastal Louisiana [14]. However, season and not site influenced the composition of the bacterial microbiome of *C*. *virginica* in the Long Island Sound Estuary [15]. *C*. *virginica* in the Chesapeake Bay were found to have microbiota dominated by members of the Pelagibacteraceae and genus *Synechococcus* [16], both common groups of bacteria in marine plankton, while other studies suggest that filter feeders contain tissue, mantle, and stomach microbiota that are distinct from the microbial composition of the overlying water column [17,18]. A number of microbiome studies have also described *Crassostrea gigas*, the Pacific Oyster. It has been found that gut microbiota within *C*. *gigas* differed more by host genotype than by geographic separation [19]. Lokmer et al. observed that the relative contributions of environmental and host genetic influences on the hemolymph microbiome depend on scale, with high microbiome variability observed even at small scale, likely driven by host genetics [20]. Trabal et al. also found that site had an influence on gut microbiota recruitment, with the caveat that greater variability was observed between oyster larval and adult life stages [21]. It has been observed that, with the exception of severely affected oysters, heat shock disrupts the microbiome principally at the OTU level and changes at higher taxonomic level were not observed. Challenging stressed individuals with potential bacterial pathogens did not produce a significant increase in disease or in the abundance of pathogenic taxa, again, except for the most affected individuals. This could be an indication of functional redundancy within the bivalve microbiome and could indicate a key role for microbiota in host health [19, 22]. This functional role is further supported by the finding that disease-susceptible oysters contain significantly different microbiota than disease-resistant individuals [23].

Compared to the marine *Crassostrea*, there is relatively little research into the microbiota of freshwater bivalves. The different physicochemical conditions of the freshwater environment could generate different selection pressures for the recruitment of bacterial taxa. Freshwater mussels present an ideal study system for the mechanisms of microbiome recruitment as they typically occur in dense and speciose aggregations that are distributed patchily throughout a river [10,24]. Recent research has shown differences in bacterial composition between clams, oysters, and mussels, which mirrored differences in the rates of organic matter processing, which could imply a strong functional role of the microbiome in host metabolism [25]. However, the present research is the first to our knowledge to characterize the gut microbiota of closely related freshwater mussel species.

In this study, we explored whether site and host species are factors influencing the composition of riverine mussel microbiota, and whether those microbiota are selectively retained from filtered particle-bound bacterial assemblages. Our study examined the gut bacterial communities of four freshwater mussel species (family Unionidae) native to the Sipsey River, Alabama, in the Gulf region of the United States. *Cyclonaias asperata*, *Fusconaia cerina*, *Lampsilis ornata*, and *Obovaria unicolor* are found throughout the Mobile River system and the Sipsey River [1], which, unlike many other southeastern US rivers, still supports diverse mussel assemblages [26]. We hypothesized that the gut bacterial community of freshwater mussels in the Sipsey River would differ significantly from the freely suspended bacteria in the water column as a result of host species recruitment of potentially beneficial taxa. Our results demonstrate that: (1) freshwater mussels harbor microbiota that are significantly different in diversity, composition and structure than those on freely suspended seston; (2) there are significant differences in the relative abundances of different bacterial taxa between co-occurring mussel species; (3) site is a significant factor in the composition of the gut microbiome within three of the four mussel species, although the influence of site is less than that of species.

## Materials and methods

**Fig 1:**
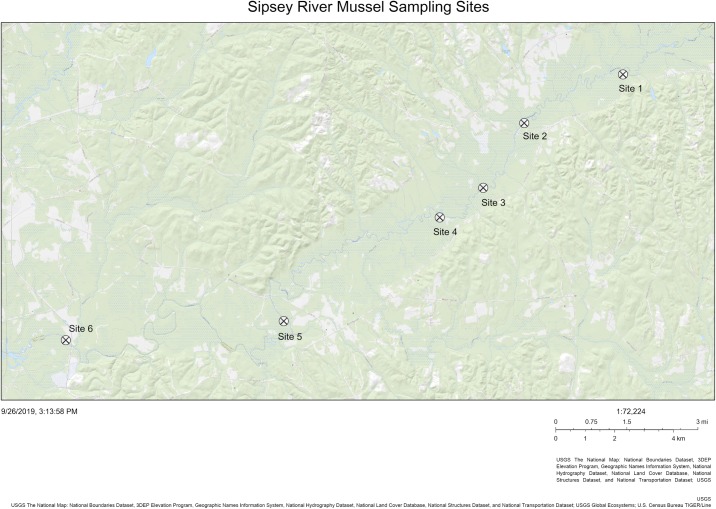
Five individuals each of four mussel species and three samples of seston were collected between July 28 and September 16, 2016 from each site shown along a ~50 km stretch of the Sipsey River, Alabama, USA. Temperature, pH, specific conductance (μS/cm), conductivity (μS/cm), dissolved oxygen (mg/L), and ammonia, orthophosphate, nitrate, and nitrite from both the water column and the sediment (μg/L) were collected at three times from each site between June 9 and September 28, 2016. The river flow is from northeast to southwest.

We sampled six sites along a ~50 km stretch of the Sipsey River in western Alabama, USA between July 28 and September 16, 2016. From upstream to downstream, the sites were identified as Site 1, Site 2, Site 3, Site 4, Site 5, and Site 6 (Fig 1). Samples sites were located on private land with access for sampling being granted by the landowners to CLA. Physicochemical parameters including temperature, pH, specific conductance (μS cm^-1^), conductivity (μS cm^-1^), and dissolved oxygen (mg L^-1^) were measured using a calibrated multiparameter sonde (YSI Inc., Yellow Springs, OH) during each collection period. We also sampled and filtered (47-mm GF/F; 0.7 μm pore size; EMD Millipore, Buckinghamshire, U.K.) surface and porewater ammonia (μg L^-1^), orthophosphate (μg L^-1^), nitrate (μg L^-1^), and nitrite (μg L^-1^) at all sites between June and September 2016. Water samples were analyzed for ammonia and orthophosphate using a Lachat QuikChem FIA +8000 Series flow injection analyzer (Hach Company, Loveland, CO, U.S.A.).

Five individuals of each of four mussel species (*C*. *asperata*, *F*. *cerina*, *L*. *ornata*, and *O*. *unicolor*) were collected from each site, with the exceptions of Site 5 where only three individuals of *F*. *cerina* were recovered, and Site 1 where no individuals of *O*. *unicolor* were found. Mussels were collected under the authority of permit 2016077745468680 issued by the Alabama Department of Conservation and Natural Resources to CLA. Length and live weight of individual mussels were determined in the field, and mussels were transported live, maintained in a cooler with moist towels to the University of Alabama for processing (travel time 45–60 minutes). Sterile knives were then used to remove the whole gut which was placed in a sterile 4.0 mL cryogenic vial and frozen (-80°C). Frozen gut samples were shipped overnight on dry ice to the University of Mississippi for subsequent DNA extraction. Three samples of seston were collected at each site by filtering 100 mL of river water through 47 mm, 1 μm pore size glass-fiber filters which were placed in sterile 5 ml DNA bead tubes from a MoBio PowerWater DNA Isolation Kit (MoBio, Carlsbad, CA) and stored frozen before shipment with the mussel samples.

DNA was extracted using a MoBio PowerSoil DNA Isolation Kit (MoBio, Carlsbad, CA). A pilot study found that using a large amount of gut tissue in the extraction process inhibited downstream amplification, and five samples (two from *C*. *asperata*, two from *F*. *cerina*, and one from *L*. *ornata*) were lost while refining the procedure. The final extraction procedure first removed gut contents from tissue by repeatedly washing the gut with extraction buffer from the DNA Isolation kit. A sterile 200 μL pipet was gently inserted into the gut and the buffer was slowly expelled into the tissue. The liquid that eluted out was pipetted back in and the process repeated ten times to isolate gut contents from tissue. The final effluent, consisting of gut contents and associated microbiota was targeted for DNA extraction following the standard extraction protocol.

Bacterial DNA was amplified twice, targeting the V4 region of the 16S rRNA gene. Nearly the entire 16S rRNA gene was first amplified using the bacteria specific Bac8f primer (5’-AGAGTTTGATCCTGGCTCAG-3’) and the universal Univ1492r primer (5’-GGTTACCTTGTTACGACTT-3’) using 0.2 mM deoxyribonucleotide triphosphates (dNTPs), 0.4 μM of each primer, 1.25 U of *Taq* polymerase, and a buffer composed of 1.5 mM MgCl_2,_ 10mM Tris-HCl, 50 mM KCl, and 0.1% Triton X-100, as follows: initial denaturation 2 min at 95°C, followed by 26 cycles of 95°C (1 min), 45°C (1 min), and 72°C (2 min), ending with a final extension at 72°C for 7 min [27]. The second amplification targeted the V4 region using dual-indexed barcoding and the primers and procedures of Kozich et al. [28]. One microliter of amplified product from the first reaction was combined with 1 μL of each barcoded primer (10 μM concentration) and 17 μL of AccuPrime *Pfx* SuperMix (Life Technologies Corporation, Carlsbad, CA). The second amplification consisted of 95°C for 2 min, followed by 30 cycles of 95°C (20 s), 55°C (15 s), 72°C (2 min), and a final elongation at 72°C for 10 min [28,29]. Amplicon concentration was normalized using a SequalPrep^TM^ Normalization Plate Kit (Invitrogen Corporation, Carlsbad, CA), and the amplified 16S rRNA gene fragments were sequenced using 251x251 PE reads on the Illumina MiSeq platform at Molecular and Genomics Core Facility of the University of Mississippi Medical Center (UMMC).

Illumina sequence data (FASTQ files) were processed using mothur [30] following the pipeline suggested by Schloss et al. [31] and Kozich et al. [28]. Sequences were aligned against the Silva database release 132 [32] and classified against version 16 of the RDP 16 database [33]. Sequences attributed to chloroplasts, mitochondria, Archaea, Eukarya, or unclassified were removed, as were sequences that were potential chimeras. Sequence data was rarefied to 5,527 sequences and six samples (four of *F*. *cerina* and two of *C*. *asperata*) with <5,000 reads and were removed from the dataset to more accurately assess microbiome diversity. Thus, the final dataset consisted of 102 total mussel samples with the species counts: *C*. *asperata* (n = 26), *F*. *cerina* (n = 22), *L*. *ornata* (n = 29), *O*. *unicolor* (n = 25), and the site counts: Site 1 (n = 15), Site 2 (n = 19), Site 3 (n = 20), Site 4 (n = 17), Site 5 (n = 17), and Site 6 (n = 14). All 18 seston (three from each site) samples were sequenced successfully and yielded sufficient reads for our analyses. Bacterial sequences from all samples were grouped into operational taxonomic units (OTUs) based on 97% similarity. OTUs represented by <0.01% of the total recovered sequences [29], in this case ≤4 sequence reads, were removed prior to beta diversity analyses.

To assess alpha diversity, Shannon’s Index was used to calculate community evenness, Chao1 was used for species richness, and the Inverse Simpson index was used for calculating diversity. Student’s t-tests were used to determine if mean coverage differed between seston and mussel samples. One-way ANOVAs were used to determine whether species evenness, richness, and diversity varied by species with seston included as a group. Separate two-way ANOVAs were performed to calculate alpha diversity differences between species and sites with seston removed as a group. MANOVA was used to determine whether there were significant differences in the relative abundances of major phyla and abundant genera between mussel gut samples and the overlying seston. The abundance-based Bray-Curtis index was used to identify structural differences between the bacterial inhabitants of the seston and mussel gut communities. A permutational multivariate analysis of variance (PERMANOVA) test was used to determine which sites/species differed significantly in the dissimilarity matrix. The Bray-Curtis dissimilarities were visualized using non-metric multidimensional scaling (NMDS) ordinations derived using the metaMDS function in the Vegan package [34], in R version 3.6.1. To determine which OTUs were most critical in driving differences seen in the dissimilarity data, Spearman’s rank correlation was used to derive effect sizes as measures of importance. Effect sizes of ≥0.75 were considered large enough for taxa to be important. The corr.axes function in mothur was used to derive effect sizes as well as coordinates of critical OTUs which could be overlaid as vectors on NMDS ordinations. The core microbiomes of the mussel species were defined as those taxa present at a ≥0.1% relative abundance in all samples of a given species. These were calculated using the Get.coremicrobiome function in mothur. Principal coordinates analysis was used to determine if bacterial composition of either the seston or mussel gut were influenced by physicochemical parameters in the river. Statistical analyses were performed in R version 3.6.1 [35].

Data: Sequence data has been deposited in the NCBI Sequence Reads Archive under the overall accession number PRJNA574208 and individual BioProject numbers 449393 (seston samples) and 1775950 (mussel gut samples).

## Results

**Fig 2:**
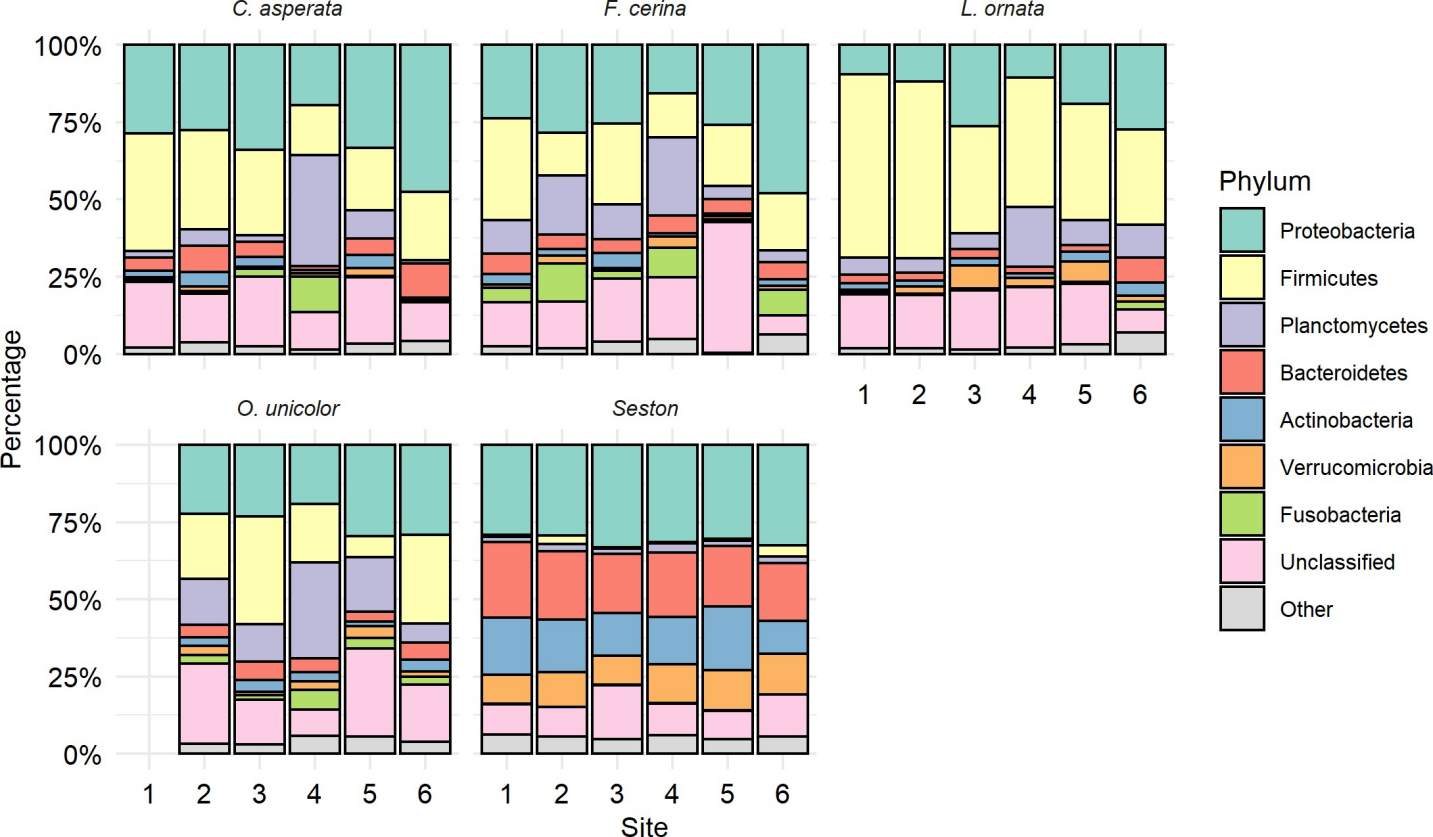
Major bacterial phyla in the gut microbiome of four freshwater mussel species (in order shown: *Cyclonaias asperata*, *Fusconaia cerina*, *Lampsilis ornata*, and *Obovaria unicolor*) at six sites in the Sipsey River, AL, USA, and for suspended seston collected from the same sites, as determined by Illumina 16S rRNA gene sequencing. Stacked bar plots are arranged with the most abundant phylum overall (Proteobacteria) at the top. No *O*. *unicolor* samples were found at site 1. The seven most abundant phyla are shown and represent 80% of all sequencing reads. Unclassified sequences made up 16% of the total dataset. All other bacterial phyla are grouped together as “other.” Each of the phyla shown differed significantly in relative abundance between the mussel gut and overlying seston with the exception of Proteobacteria.

The final dataset consisted of 3,722,899 total sequences, of which 142,827 were unique. Seven bacterial phyla accounted for 80% of all sequence reads: Proteobacteria (25.9%), Firmicutes (22.9%), Planctomycetes (10.9%), Bacteroidetes (8.0%), Actinobacteria (5.4%), Verrucomicrobia (4.0%), and Fusobacteria (2.7%), with an additional 16.2% unclassified at the phylum level (Fig 2). Except for Proteobacteria (MANOVA, p = 0.18, F = 1.88), the relative abundance of the major phyla differed significantly between mussel and seston samples, with Firmicutes (p<0.001, F = 25.13), Plactomycetes (p = 0.02, F = 5.83), and Fusobacteria (p = 0.05, F = 4.35) making up a larger percentage of the mussel microbiome and Bacteroidetes (p<0.001, F = 243.62), Actinobacteria (p<0.001, F = 233.5), and Verrucomicrobia (p<0.001, F = 128.43) being more abundant in the seston. Within the Proteobacteria, 41% of Proteobacterial sequences belonged to the Alphaproteobacteria, while Gammaproteobacteria (36.4%), Betaproteobacteria (17.6%), Deltaproteobacteria (2.3%), and Epsilonproteobacteria (0.3%) accounted for the rest of the Proteobacteria. Alphaproteobacteria and Gammaproteobacteria were similarly abundant between mussel species and seston, although the abundance of Gammaproteobacteria was significantly lower in *L*. *ornata* than in any other species or seston (MANOVA, p = 0.002, F = 4.63). Betaproteobacteria, however, was significantly higher in abundance in seston than in any mussel species (MANOVA, p<0.001, F = 47.06).

**Fig 3:**
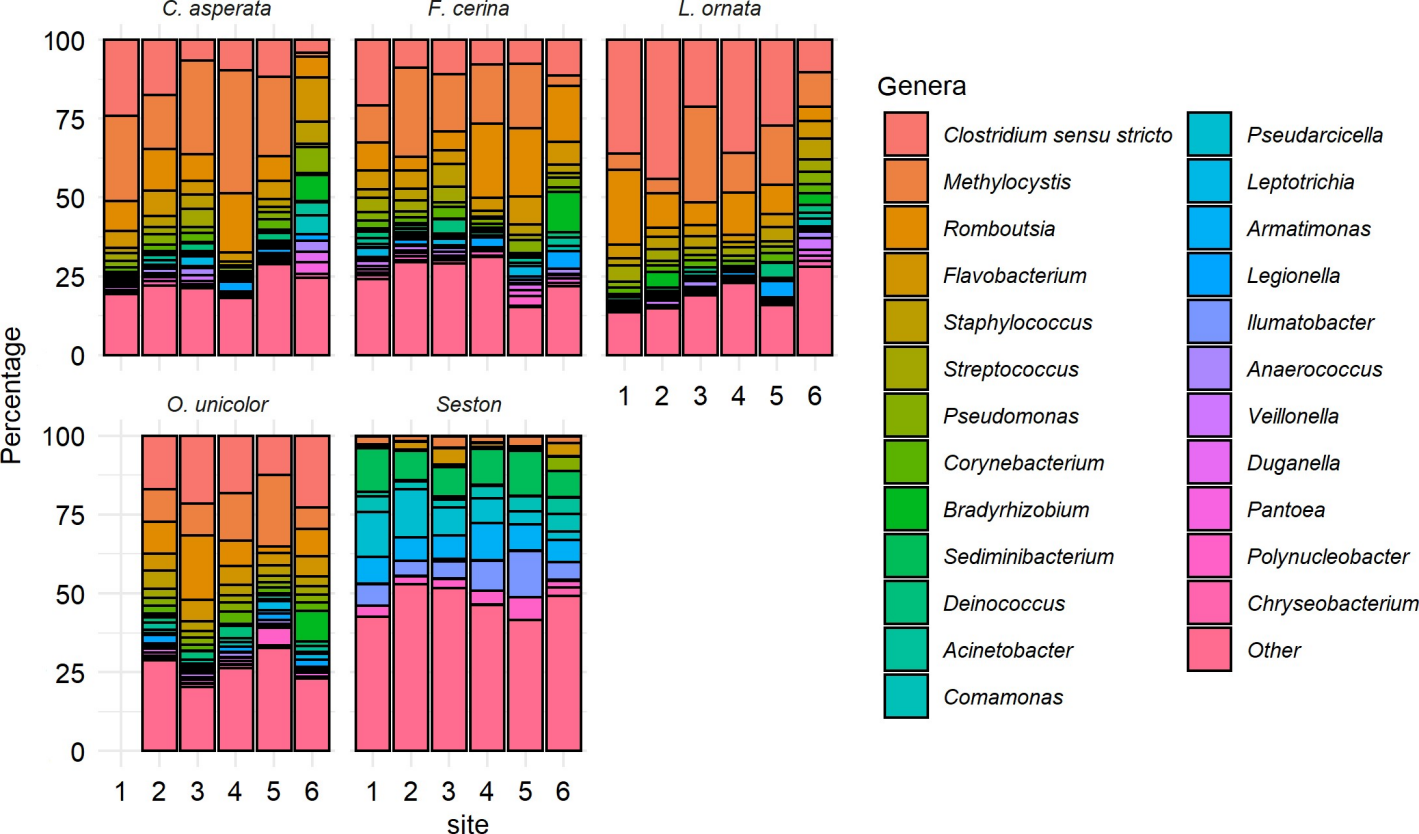
Major bacterial genera in the gut microbiome of four freshwater mussel species (in order shown: *Cyclonaias asperata*, *Fusconaia cerina*, *Lampsilis ornata*, and *Obovaria unicolor*) at six sites in the Sipsey River, AL, USA, and for suspended seston collected from the same sites, as determined by Illumina 16S rRNA gene sequencing. Stacked bar plots are arranged with the most abundant genus overall (*Clostridium sensu stricto*) at the top. No *O*. *unicolor* samples were found at site 1. The 24 most abundant genera are shown and represent 73% of all sequencing reads that were identified to the genus level. Sequences classified at the genus level made up 35.7% of the total data. All other bacterial genera are grouped together as “other”.

At a finer taxonomic scale, 1,327,260 of the 3,722,899 (35.7%) total retained sequences in the dataset were classified down to the genus level. Sequences grouped into 699 defined genera; of these, 24 genera contained > 10,000 sequences and together accounted for 73.7% of the total sequences classified at the genus level. *Clostridium* was the most abundant named genus (211,068 sequences, 15.9% relative abundance of the named sequences), followed by *Methylocystis* (12.6%), *Romboutsia* (9.5%), *Flavobacterium* (5.8%), *Staphylococcus* (3.2%), *Streptococcus* (2.8%), *Pseudomonas* (2.5%), *Corynebacterium* (2.0%), *Bradyrhizobium* (1.7%), and *Sediminibacterium* (1.6%) (Fig 3). With the exceptions of *Bradyrhizobium*, *Acinetobacter*, and *Chryseobacterium*, each of the major genera differed significantly in relative abundance between seston and mussels. Most of the major genera were more abundant in the mussel gut than suspended on seston, with the exceptions of *Sediminibacterium*, *Comamonas*, *Pseudarcicella*, *Armatimonas*, *Ilumatobacter*, and *Polynucleobacter*.

**Fig 4:**
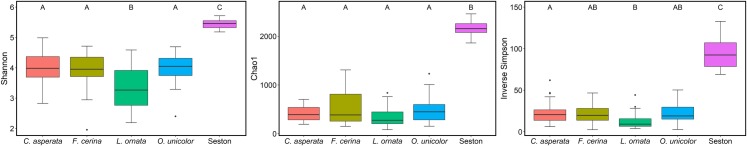
Shannon evenness, Chao1 richness, and Inverse Simpson diversity of the gut microbiota of four freshwater mussel species (in order shown: *Cyclonaias aspertata*, *Fusconaia cerina*, *Lampsilis ornata*, and *Obovaria unicolor)* at six sites in the Sipsey River, AL, USA, and for seston collected from the same sites, as determined from Illumina 16S rRNA gene sequencing. Seston bacterial evenness, richness, and diversity were higher than those metrics observed in the mussel gut (p < 0.001). *L*. *ornata* the lowest evenness and diversity scores of the four mussel species. Error bars represent standard error of the 22–29 samples of each mussel species collected, or the 18 seston samples collected across all sites.

Sequences grouped into 11,013 OTUs. Seven OTUs were represented by >100,000 sequences each and together accounted for 30% of the total reads. The most abundant OTU was classified as a member of the order Clostridiales (Phylum: Firmicutes) and accounted for 8.3% of all sequence reads obtained, while the second most abundant OTU (5.1% of reads) was identified as genus *Clostridium* in the same order. The third most abundant OTU (4.5% of reads) was classified within genus *Methylocystis* (Alphaproteobacteria), and other dominant OTUs included two members of the Planctomycetaceae (Planctomycetes; accounting for 3.7% and 3.1% of reads), *Romboutsia* (Firmicutes; 3.4% reads), and an unclassified bacterium (2.8% reads). Mean coverage of mussel samples was 0.98, while coverage of the seston samples was 0.89. Bacterial communities associated with seston were significantly more even (Shannon, p<0.001), diverse (Inverse Simpson, p<0.001), and presented a higher richness (Chao1, p<0.001), than any mussel-associated communities. Each of the four mussel species was similar in the three alpha diversity metrics, although *L*. *ornata* was significantly less even than the other three species and also had the lowest diversity (Fig 4). Because seston had significantly higher alpha diversity than any of the mussel species, metrics were re-calculated with seston removed. For both Shannon evenness (p<0.001, F = 2.54, ANOVA) and Inverse Simpson diversity (p = 0.002, F = 5.58) species had a significant effect with *L*. *ornata* having lower diversity than the other three species. Across all three metrics, site did not have a significant effect (p between 0.356 and 0.692, F between 0.611 and 1.122).

**Fig 5:**
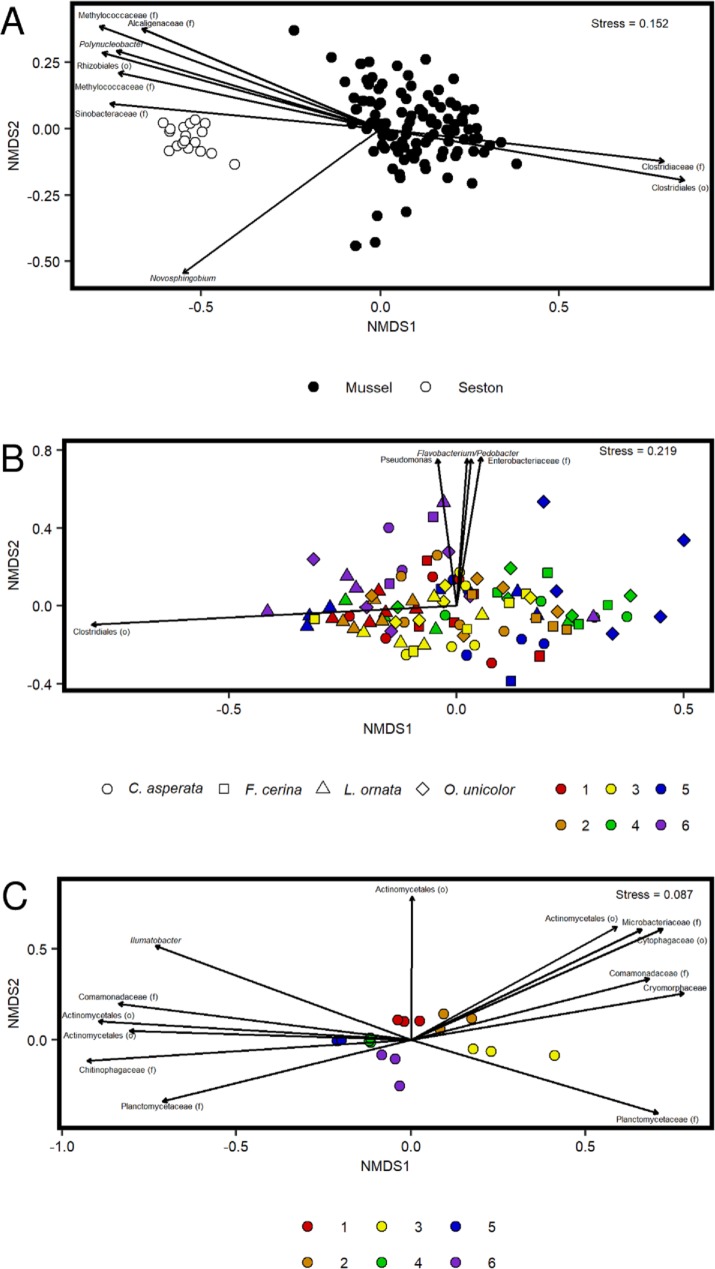
NMDS ordinations of 16S rRNA bacterial microbiome data collected from the mussel species *Cyclonaias asperata*, *Fusconaia cerina*, *Lampsilis ornata*, and *Obovaria unicolor* from the Sipsey River, AL, USA, in addition to suspended seston. Important OTUs driving the ordination are indicated with an arrow, with arrow length proportional to effect size and arrow direction reflecting association with those samples. **A.** Bray-Curtis ordination of samples categorized by associated environment (mussel vs. seston). **B.** Bray-Curtis ordination of mussel samples only. Species are represented by shape and sites are represented by colors. **C.** Bray-Curtis ordination of seston samples only. Sites are represented by colors.

A Bray-Curtis dissimilarity matrix clearly separated samples according to whether contents were mussel- or seston-associated (Fig 5A) and each pairwise comparison of mussel species to seston was significant (PERMANOVA, p<0.01 for all, F between 22.8 and 32.9). Thus, seston and mussel samples were analyzed separately to determine if there were site and/or species-specific differences. Bacterial composition differed significantly between all pairs of mussel species (Table 1) except for *F*. *cerina* and *O*. *unicolor* (PERMANOVA, p = 0.43, F = 3.64). Site also influenced mussel microbiome composition, although the effect was less observable than the effect of species. Thirteen of 16 pairwise site comparisons were significant when all samples were assessed together (Table 1). The only non-significant pairwise comparisons were between sites 1 and 2, 2 and 5, and 2 and 6. This pattern was apparent in the mussel only NMDS (Fig 5B) with most of the separation by species and site 2 clustering toward the center with the other sites radiating outward. Because it was possible that species differences could confound compositional differences between sites, four separate Bray-Curtis distance matrices were derived for each of the four mussel species individually. One-way PERMANOVAs found that for *F*. *cerina* (p = 0.011, F = 1.943), *L*. *ornata* (p<0.001, F = 3.171), and *O*. *unicolor* (p<0.001, F = 2.886) the effect of site was significant, while for *C*. *asperata* (p = 0.089, F = 1.506) there was no significant difference between sites. Although the overall effect of site was significant in the seston-associated bacterial samples (p < 0.001), none of the pairwise comparisons between sites were significant, although sites did tend to cluster in NMDS ordination (Fig 5C). Overall, species had a greater effect on the mussel gut microbiome than site, and the mussel gut microbiome was distinct from that of the surrounding seston.

**Table 1:** Pairwise species and site comparisons for gut microbiomes of freshwater mussels collected from six sites in the Sipsey River, AL, USA. Microbiome comparisons were based on the Bray-Curtis dissimilarity metric. Mussel species are *Cyclonaias asperata*, *Fusconaia cerina*, *Lampsilis ornata*, and *Obovaria unicolor* and the six sites cover a ~50 km stretch of the river, numbered from upstream to downstream. P-values come from a PERMANOVA comparing bacterial community composition by host mussel species and by site. The test excluded seston samples which were significantly different from each mussel species at each site (p <0.01 for all). The R package pairwiseAdonis was used to perform a post-hoc test to determine which pairwise combinations of species and site were significant. Adjusted p-values of this post-hoc test are displayed below.

Species Comparisons		Site Comparisons	
Species	p-value	Sites	p-value
*C*. *asperata–F*. *cerina*	0.012	1–2	0.780
*C*. *asperata–L*. *ornata*	0.006	1–3	0.015
*C*. *asperata–O*. *unicolor*	0.006	1–4	0.015
*F*. *cerina–L*. *ornata*	0.006	1–5	0.015
*F*. *cerina–O*. *unicolor*	0.240	1–6	0.015
*L*. *ornata–O*. *unicolor*	0.012	2–3	0.015
		2–4	0.045
		2–5	0.660
		2–6	0.150
		3–4	0.015
		3–5	0.030
		3–6	0.015
		4–5	0.015
		4–6	0.030
		5–6	0.015

Proportions of the two most abundant OTUs (both within order Clostridiales) were higher in the mussel gut and were important in separating mussel samples from seston samples in NMDS (Fig 5A). The most abundant of these OTUs accounted for at least 1% of the sequences recovered from 92 of the final 102 mussel samples. A core microbiome analysis showed that none of the OTUs were present at a relative abundance of 0.1% across all of the mussel samples. Thus, the core microbiome analysis was repeated for each of the four mussel species individually. For *C*. *asperata*, OTUs 1, 2, 3, 5, and 12 were present at 0.1% abundance across all replicates of the species, representing a Clostridiales, Clostridiaceae, *Methylocystis*, *Romboutsia*, and *Staphylococcus*, respectively. The core microbiome of *F*. *cerina* consisted of OTUs 1, 2, and 7, a taxon unclassified at the phylum level. The core microbiome of *L*. *ornata* consisted only of OTU 12, a *Staphylococcus*. The core microbiome of *O*. *unicolor* consisted of OTUs 2 and 3, Clostridiaceae and *Methylocystis*. OTUs associated with seston included representatives from the Methylococcaceae, *Polynucleobacter*, and Rhizobiales. The Clostridiales OTUs were also important in separating mussel microbiome samples by species, being associated with *L*. *ornata* in particular (Fig 5B). Other OTUs that were important in separating mussel samples were associated with a subset of mussels collected from sites 5 and 6, and included a representative of the Enterobacteriaceae as well as OTUs from the genera *Flavobacterium*, *Pedobacter*, and *Pseudomonas*. Seston samples clustered by site (Fig 5C), with adjacent sites generally being more similar (an exception being sites 3 and 4). Various OTUs were associated with separation of seston samples but four OTUs (classified as members of the Cytophagaceae, Comamonadaceae, *Sediminibacterium*, and *Armatimonas*) each accounted for at least 1% of the sequence reads obtained from every seston sample.

**Fig 6:**
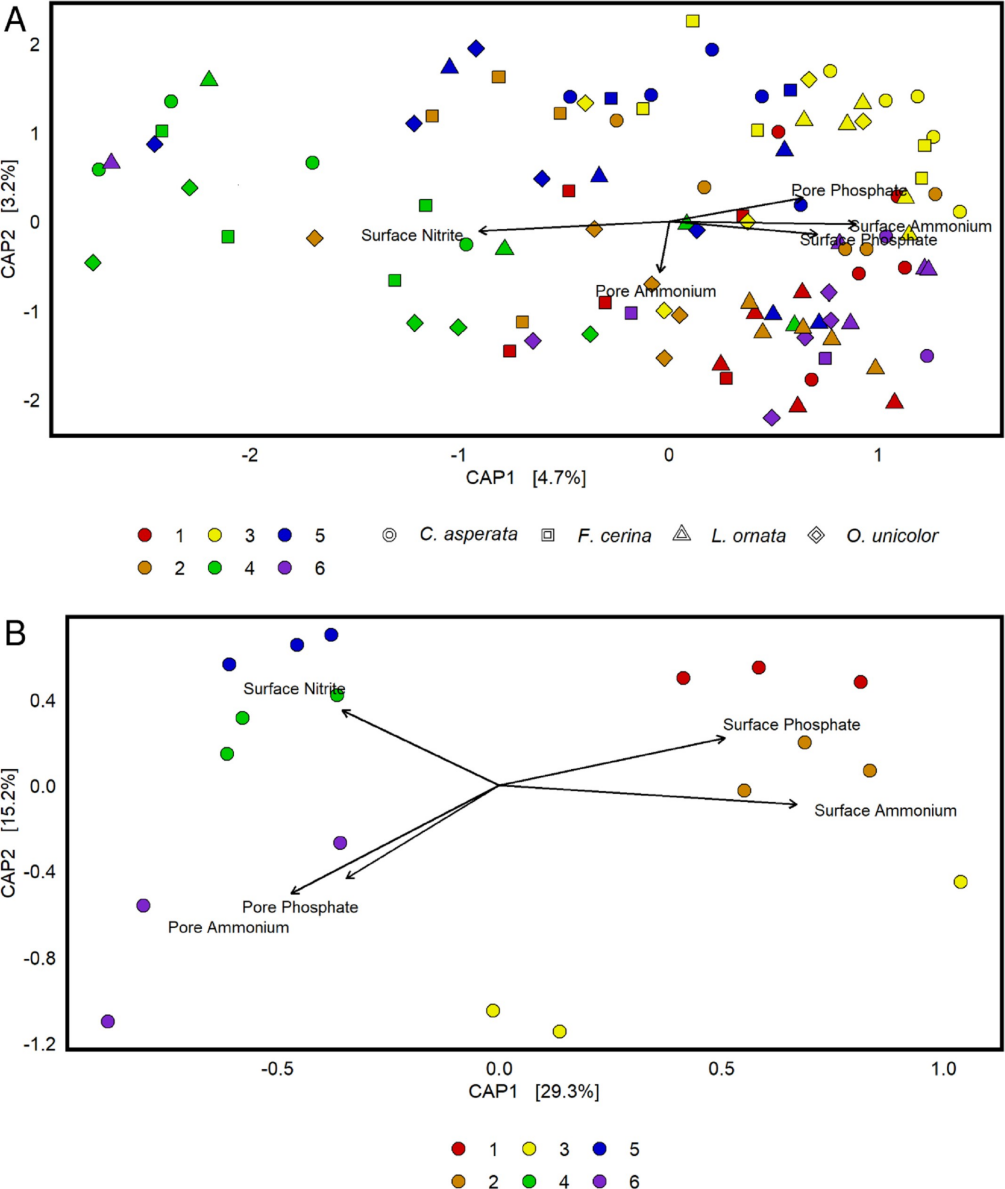
A constrained principal coordinates analysis (CAP) of 16S rRNA bacterial microbiome data collected from the mussel species *Cyclonaias asperata*, *Fusconaia cerina*, *Lampsilis ornata*, and *Obovaria unicolor* as well as seston from the Sipsey River, AL, USA. Bray-Curtis dissimilarity was used to separate samples by site differences in physicochemical parameters. Three measurements of each environmental parameter were recorded between June 9 and September 28, 2016. CAP identified surface water ammonium, porewater ammonium, surface phosphate, pore phosphate, and surface nitrite as the most critical factors of all the measured parameters and those factors were retained in a reduced model. Surface measurements were collected from the flowing water body and pore measurements were taken from pore water. A. Within the mussel gut samples, these physicochemical parameters explained very little variability, combining 7.9% across each of the first two axes. B. Within the seston samples, environmental chemistry explained 44.5% of bacterial compositional differences across the two axes.

Sites differed in their physicochemistry (Table 2). Principal coordinates analysis ranked surface water ammonium, porewater ammonium, surface phosphate, pore phosphate, and surface nitrite as the most critical factors in separating bacterial composition between sites, and those factors were retained in a reduced model (Fig 6). It was clear that site physicochemistry had a greater effect on the seston bacterial community than on the mussel gut community as the first two axes of the PCoA explained 44.5% of the seston variability and only 7.9% of the mussel gut variability. Higher average values for surface ammonium and phosphate were observed at sites 1–3 which separated distinctly from sites 4–6 along the x-axis (Fig 6B). Site 6 was particularly high in pore ammonium and pore phosphate while sites 4 had the highest levels of surface nitrite.

**Table 2:** Physicochemical data from six sites along the Sipsey River, AL, USA. “Surface” measurements reflect readings from the water column and “pore” reflects porewater readings. Sites are ordered moving downstream and the distance from the first site is noted. Three measurements of each environmental parameter were recorded between June 9 and September 28, 2016. Data shown reflects the means of these three measurements and the standard error.

Metric	Sample site					
	1	2	3	4	5	6
Distance (km)	0	7.2	10.7	14.0	27.4	44.6
Surface ammonia (μg L^-1^)	24.3 ± 0.6	19.7 ± 1.3	21.1 ± 0.3	8.4 ± 0.1	18.5 ± 0.1	17.5 ± 0.4
Pore ammonia (μg L^-1^)	127.5 ± 22.6	269.0 ± 171.8	164.3 ± 70.6	268.7 ± 55.4	117.1 ± 65.5	501.0 ± 228.1
Surface orthophostate (μg L^-1^)	7.3 ± 1.1	5.2 ± 0.4	4.8 ± 0.2	2.1 ± 0.2	5.8 ± 0.3	4.8 ± 0.1
Pore orthophosphate (μg L^-1^)	8.0 ± 1.4	6.1 ± 0.8	15.3 ± 7.9	1.2 ± 0.3	17.9 ± 8.8	20.3 ± 12.3
Surface nitrite (μg L^-1^)	4.5 ± 0.3	2.7 ± 0.2	2.0 ± 0.2	15.9 ± 0.8	3.1 ± 0.1	2.4 ± 0.2
Pore nitrite (μg L^-1^)	4.3 ± 1.0	3.0 ± 1.4	1.3 ± 0.1	30.2 ± 9.0	6.2 ± 1.9	29.0 ± 26.5
Surface nitrate (μg L^-1^)	309.0 ± 19.3	309.0 ± 19.3	309.0 ± 19.3	154.8 ± 1.0	350.9 ± 0.1	201.0 ± 4.6
Pore nitrate (μg L^-1^)	10.1 ± 8.9	20.4 ± 2.5	13.7 ± 7.0	31.2 ± 8.9	40.3 ± 11.8	10.6 ± 7.2
Temperature (°C)	26.2 ± 0.5	25.7 ± 0.7	27.1 ± 0.5	27.5 ± 0.8	28.2 ± 1.0	27.4 ± 0.3
pH	7.2 ± 0.1	7.2 ± 0.0	7.2 ± 0.0	7.3 ± 0.1	7.2 ± 0.1	7.2 ± 0.1
Specific conductance (μS cm^-1^)	141.9 ± 29.6	127.5 ± 13.7	140.2 ± 9.1	134.4 ± 12.3	80.1 ± 38.9	119.8 ± 20.4
Conductivity (μS cm^-1^)	144.7 ± 29.0	130.3 ± 15.5	145.8 ± 10.8	141.3 ± 14.8	113.5 ± 20.8	122.3 ± 21.3
Dissolve oxygen (mg L^-1^)	7.0 ± 1.0	6.8 ± 0.1	5.4 ± 0.0	6.8 ± 0.1	7.4 ± 0.6	5.3 ± 0.8

## Discussion

This study demonstrated that the structure of the gut bacterial microbiome of four co-occurring freshwater unionid mussel species differed in composition from the bacterial communities of the overlying water that they filter. This has been suggested from studies in marine systems [17,18], and our results show similar differentiation in a freshwater system. Our study showed significant differences between mussel microbiome and seston even at high levels of prokaryote classification (i.e. phylum level). Firmicutes and Planctomycetes were major constituents of the mussel microbiome, while they were only a minor percentage of the overlying seston. The opposite was true for Actinobacteria and Verrucomicrobia which were significantly more abundant in the seston. This suggests that mussels are selectively retaining certain taxa in the gut, and that their gut bacterial microbiome is not just dependent on the bacteria associated with the particles that they ingest. However, Proteobacteria accounted for approximately 26% of both types of assemblages, and this high proportion of Proteobacteria in the mussel microbiome matches results from previous studies of marine bivalves [18,36,37]. In contrast, a study of the gut microbiota of nine individuals of a single freshwater mussel species (*Villosa nebulosa*) found that Proteobacteria accounted for just under 5% of the bacterial gut community [38]. However, that study had low sequencing depth (average of <4,000 sequences per individual; below the threshold for retention in our dataset) and sequences were classified to an older database, so comparisons are difficult and could reflect methodological differences. Perhaps more surprisingly, unlike previous studies which have shown an abundance of Betaproteobacteria in bivalves [21] it was found that within the Proteobacteria, Betaproteobacteria were disproportionately abundant in the seston while Alpha and Gammaproteobacteria were greater contributors to the mussel gut. The previous study also identified Alpha and Gammaproteobacteria as major gut constituents, but it is unclear why Betaproteobacteria were observably less abundant in our samples.

While Proteobacteria were proportionally abundant in the mussel microbiome, *Pseudomonas spp*. accounted for a very small proportion of the total bacterial community, making up less than 0.01% of the total bacterial community. *Pseudomonas* made up an even smaller fraction of the seston community, so there may have been some retention in the gut, but not to the levels previously described. The assumption that pseudomonads and vibrios dominate the bivalve microbiome [37,39] is being challenged by studies that have used culture-independent approaches in marine systems [22,40,41], and their low proportions here suggest the same may apply in freshwater environments. Each of the two most abundant taxa in the total dataset were Clostridiales which have not, to our knowledge, been previously reported as major freshwater bivalve gut constituents, although they are common and widespread in the gut microbiota of vertebrates [42]. These OTUs were significantly more abundant in the mussels than in the seston, again indicating selective retention by the mussel host. A core microbiome analysis found that while there was no conserved gut microbiome across the four observed mussel species, each species did have its own core microbiome. Clostridiales were core members of the microbiota of both *C*. *asperata* and *O*. *unicolor*, further supporting their potential selective retention within the gut.

Previous studies have identified nitrogen-fixing bacteria as a part of the bivalve microbiome [43,44], with evidence that these bacteria make nitrogen biologically available to the host to metabolize. The third most abundant taxon in our dataset was *Methylocystis*, a methanotrophic N_2_-fixer [45,46], and sequences of this OTU were significantly more abundant in mussels than in seston. *Methylocystis* was a member of the core microbiome in both *C*. *asperata* and *O*. *unicolor*. Whether this bacterium could provide fixed nitrogen to the host remains to be determined, but it could be a plausible explanation for its accumulation in the mussel gut compared to the surrounding water. Mussels have been shown to affect the diversity and relative abundance of nitrogen-cycling bacteria in sediment through aeration while burrowing and through the release of secondary metabolites [47]. The latter mechanism is particularly interesting, as if mussel metabolites can influence external microbial communities, it is possible that similar processes could occur within the host. Given that the highest ammonia level recorded in the sediment was 0.95 mg L^-1^, and the most susceptible unionid has a median LC50 for ammonia of 2.56 mg L^-1^ [48], it is unlikely that any of the mussels in this study were under ammonia stress, and the accumulation of biologically available nitrogen would be favorable. However, drawing a link between environmental nutrients, gut flora, and bivalve host will require further analysis of gut nutrient dynamics to determine if any fixed nitrogen is actually used by the host species.The microbiota of both the seston and mussels varied by site along the river, with 13 of 16 pairwise site comparisons differing significantly. Site was a significant factor determining microbiome community composition within three of four mussel species even when assessed separately, removing between species variability. It was unclear what was driving compositional differences along the stream, particularly considering how little variation was explained by differences in site physicochemistry. Thomas et al. [18] found that microbial enrichments from oyster tissue, mantle fluid, and sediment were more similar to each other than to an enrichment from the water column. Freshwater mussels are often partially or entirely buried within the sediment [7,49], so porewater conditions may be more relevant to their gut microbiome than the overlying water column. However, while both pore ammonium and pore orthophosphate were among the five most critical environmental factors on the mussel gut community, surface ammonium explained the greatest variability. The finding that water and porewater chemistry had a minimal influence on the mussel microbiota relative to their impact on seston further implies that bacterial retention within the gut is an active and potentially functionally selective process.

Even though the four co-occurring mussel species in our study are members of the same subfamily (Ambleminae) [50] and were exposed to the same environmental conditions, their gut microbiota differed significantly. Transient microorganisms acquired from the environment can be abundant members of animal gut communities [14,51] and could contribute noise to beta diversity analysis, especially when examining the microbiota of densely, co-occurring aquatic species which could be prone to cross-over in their microbiota. Any such noise in this study was low enough that differences in microbiome composition between co-occurring mussel species were still apparent, which strengthens the argument that freshwater mussels are selectively curating certain bacterial taxa in their digestive organs, and also implies that such curation may be host species-specific. Regardless, host species and site were factors correlated with the composition of the freshwater mussel gut microbiome, even within a single mussel subfamily in a single river. The process by which mussels curate a gut microbiome and the function of these gut microorganisms to the host warrant further investigation in these important aquatic invertebrates.

## References

[1] Williams JD, Bogan AE, Garner JT. Freshwater mussels of Alabama and the Mobile basin in Georgia, Mississippi, and Tennessee Tuscaloosa: University of Alabama Press; 2008

[2] Neves RJ, Bogan AE, Williams JD, Ahlstedt SA, Hartfield PW. Status of aquatic mollusks in the southeastern United States: a downward spiral of diversity In: Benz GW, Collins DE, editors. Aquatic fauna in peril: the southeastern perspective. Decatur: Southeast Aquatic Research Institute, Lenz Design and Communications; 1997 pp. 44–86.

[3] Parmalee PW, Bogan AE. Freshwater mussels of Tennessee. Knoxville: University of Tennessee Press; 1998.

[4] Strayer DL, Downing JA, Haag WR, King TL, Layzer JB, Newton TJ, et al Changing perspectives on pearly mussels, North America's most imperiled animals. Biosci. 2004;54: 429–439.

[5] Dudgeon D, Arthington AH, Gessner MO, Kawabata Z, Knowler DJ, Lévêque C, et al Freshwater biodiversity: importance, threats, status and conservation challenges. Biol Rev. 2006;81: 163–182. 10.1017/S1464793105006950 16336747

[6] Atkinson CL, Julian JP, Vaughn CC. Species and function lost: Role of drought in structuring stream communities. Biol Conserv. 2014;176: 30–38.

[7] Vaughn CC, Hakenkamp CC. The functional role of burrowing bivalves in freshwater ecosystems. Freshw Biol. 2001;46: 1431–1446.

[8] Atkinson CL, Sansom BJ, Vaughn CC, Forshay KJ. Consumer aggregations drive nutrient dynamics and ecosystem metabolism in nutrient-limited systems. Ecosystems. 2018;21: 521–535. PMC7252507 32461736

[9] Atkinson CL, Vaughn CC, Forshay KJ, Cooper JT. Aggregated filter‐feeding consumers alter nutrient limitation: consequences for ecosystem and community dynamics. Ecology. 2013;94: 1359–1369. 10.1890/12-1531.1 23923499

[10] Atkinson CL, Vaughn CC. Biogeochemical hotspots: temporal and spatial scaling of the impact of freshwater mussels on ecosystem function. Freshw Biol. 2015;60: 563–574.

[11] Peiffer JA, Spor A, Koren O, Jin Z, Tringe SG, Dangle JL, et al Diversity and heritability of the maize rhizosphere microbiome under field conditions. Proc Natl Acad Sci USA. 2013;110: 6548–6553. 10.1073/pnas.1302837110 23576752 PMC3631645

[12] Edwards J, Johnson C, Santos-Medellín C, Lurie E, Podishetty NK, Bhatnagar S, et al Structure, variation, and assembly of the root-associated microbiomes of rice. Proc Natl Acad Sci USA. 2015;112; E911–E920. 10.1073/pnas.1414592112 25605935 PMC4345613

[13] Rietl AJ, Overlander ME, Nyman AJ, Jackson CR. Microbial community composition and extracellular enzyme activities associated with *Juncus roemerianus* and *Spartina alterniflora* vegetated sediments in Louisiana saltmarshes. Microb Ecol. 2016;71: 290–303. 10.1007/s00248-015-0651-2 26271740

[14] King GM, Judd C, Kuske CR, Smith C. Analysis of stomach and gut microbiomes of the eastern oyster (*Crassostrea virginica*) from coastal Louisiana, USA. PLoS One. 2012;7: 1–11. 10.1371/journal.pone.0051475 23251548 PMC3520802

[15] Pierce ML, Ward JE, Holohan BA, Zhao X, Hicks RE. The influence of site and season on the gut and pallial fluid microbial communities of the eastern oyster, *Crassostrea virginica* (Bivalvia, Ostreidae): community-level physiological profiling and genetic structure. Hydrobiologia. 2016;765: 97–113.

[16] Ossai S, Ramachandran P, Ottesen A, Reed E, DePaola A, Parveen S. Microbiomes of American Oysters (*Crassostrea virginica*) harvested from two sites in the Chesapeake Bay. Genome Announc. 2017;5: e00729–17. 10.1128/genomeA.00729-17 28751404 PMC5532842

[17] Chauhan A, Wafula D, Lewis DE, Pathak A. Metagenomic assessment of the Eastern oyster-associated microbiota. Genome Announc. 2014;2: e01083–14. 10.1128/genomeA.01083-14 25342691 PMC4208335

[18] Thomas JC, Wafula D, Chauhan A, Green SJ, Gragg R, Jagoe C. A survey of deepwater horizon (DWH) oil-degrading bacteria from the Eastern oyster biome and its surrounding environment. Front Microbiol. 2014;5: 149 10.3389/fmicb.2014.00149 24782841 PMC3988384

[19] Wegner KM, Volkenborn N, Peter H, Eiler A. Disturbance induced decoupling between host genetics and composition of the associated microbiome. BMC Microbiol. 2013;13: 252 10.1186/1471-2180-13-252 24206899 PMC3840651

[20] Lokmer A, Goedknegt MA, Thieltges DW, Fiorentino D, Kuenzel S, Baines JF, et al Spatial and temporal dynamics of Pacific oyster hemolymph microbiota across multiple scales. Front Microbiol. 2016;7: 1367 10.3389/fmicb.2016.01367 27630625 PMC5006416

[21] Trabal Fernández N, Mazón-Suástegui JM, Vázquez-Juárez R, Ascencio-Valle F, Romero J. Changes in the composition and diversity of the bacterial microbiota associated with oysters (*Crassostrea corteziensis*, *Crassostrea gigas* and *Crassostrea sikamea*) during commercial production. FEMS Microbiol Ecol. 2014;88: 69–83. 10.1111/1574-6941.12270 24325323

[22] Lokmer A, Wegner KM. Hemolymph microbiome of Pacific oysters in response to temperature, temperature stress and infection. ISME J. 2015;9: 670–682. 10.1038/ismej.2014.160 25180968 PMC4331581

[23] King WL, Siboni N, Williams NLR, Kahlke T, Nguyen KV, Jenkins C, et al Variability in the composition of Pacific Oyster microbiomes across oyster families exhibiting different levels of susceptibility to OsHV-1 μvar disease. Front Microbiol. 2019;10: 473 10.3389/fmicb.2019.00473 30915058 PMC6421512

[24] Vaughn CC, Nichols SJ, Spooner DE. Community and foodweb ecology of freshwater mussels. J North Am Benthol Soc. 2008;27: 409–423.

[25] Murphy AE, Kolkmeyer R, Song B, Anderson IC, Bowen J. Bioreactivity and Microbiome of Biodeposits from Filter-Feeding Bivalves. Microb Ecol. 2019;77: 343–357. 10.1007/s00248-018-01312-4 30612185

[26] McCullagh WH, Williams JD, McGregor SW, Pierson JM, Lydeard C. The Unionid (Bivalvia) fauna of the Sipsey River in northwestern Alabama, an aquatic hotspot. Am Malacol Bull. 2002;17: 1–15.

[27] Jackson CR, Langner HW, Donahoe-Christiansen J, Inskeep WP, McDermott TR. Molecular analysis of microbial community structure in an arsenite-oxidizing acidic thermal spring. Environ Microbiol 2001;3: 532–542. 10.1046/j.1462-2920.2001.00221.x 11578314

[28] Kozich JJ, Westcott SL, Baxter NT, Highlander SK, Schloss PD. Development of a dual-index sequencing strategy and curation pipeline for analyzing amplicon sequence data on the MiSeq Illumina sequencing platform. Appl Environ Microbiol. 2013;79: 5112–5120. 10.1128/AEM.01043-13 23793624 PMC3753973

[29] Stone BWG, Jackson CR. Biogeographic patterns between bacterial phyllosphere communities of the Southern Magnolia (*Magnolia grandiflora*) in a small forest. Microb Ecol. 2016;71: 954–961. 10.1007/s00248-016-0738-4 26883131

[30] Schloss PD, Westcott SL, Ryabin T, Hall JR, Hartmann M, Hollister EB, et al Introducing mothur: open-source, platform-independent, community-supported software for describing and comparing microbial communities. Appl Environ Microbiol. 2009;75: 7537–7541. 10.1128/AEM.01541-09 19801464 PMC2786419

[31] Schloss PD, Gevers D, Westcott SL. Reducing the effects of PCR amplification and sequencing artifacts on 16S rRNA-based studies. PLoS One. 2011;6: 1–14. 10.1371/journal.pone.0027310 PMC3237409 22194782

[32] Quast C, Pruesse E, Yilmaz P, Gerken J, Schweer T, Yarza P, et al The SILVA ribosomal RNA gene database project: improved data processing and web-based tools. Nucleic Acids Res. 2013;41: D590–D596. 10.1093/nar/gks1219 23193283 PMC3531112

[33] Maidak BL, Cole JR, Lilburn TG, Parker CT Jr, Saxman PR, Stredwick JM, et al The RDP (ribosomal database project) continues. Nucleic Acids Res. 2000;28, 173–174. 10.1093/nar/28.1.173 10592216 PMC102428

[34] Oksanen J, Kindt R, Legendre P, O’Hara B, Simpson GL, Solymos P. et al The vegan package, community ecology package 2008;10: 631–637.

[35] Team RC. R: A language and environment for statistical computing. 2013

[36] Cao R, Xue CH, Liu Q, Xue Y. Microbiological, chemical, and sensory assessment of Pacific Oysters (*Crassostrea gigas*) stored at different temperatures. Czech J Food Sci. 2009;27: 102–108.

[37] Olafsen JA, Mikkelsen HV, Giæver HM, Hansen GH. Indigenous bacteria in hemolymph and tissues of marine bivalves at low temperatures. Appl Environ Microbiol. 1993;59: 1848–1854. 16348962 10.1128/aem.59.6.1848-1854.1993 PMC182171

[38] Aceves AK, Johnson P, Bullard SA, Lafrentz S, Arias CR. Description and characterization of the digestive gland microbiome in the freshwater mussel *Villosa nebulosa* (Bivalvia: Unionidae). J Molluscan Stud. 2019;84: 240–246.

[39] Miller WA, Miller MA, Gardner IA, Atwill ER, Byrne BA, Jang S, et al *Salmonella spp*., *Vibrio spp*., *Clostridium perfringens*, and *Plesiomonas shigelloides* in Marine and Freshwater Invertebrates from Coastal California Ecosystems. Microb Ecol. 2006;52: 198–206. 10.1007/s00248-006-9080-6 16897302

[40] Green TJ, Siboni N, King WL, Labbate M, Seymour JR, Raftos D. Simulated marine heat wave alters abundance and structure of *Vibrio* populations associated with the Pacific Oyster resulting in a mass mortality event. Microb Ecol. 2019;77: 736–747. 10.1007/s00248-018-1242-9 30097682

[41] King WL, Jenkins C, Go J, Siboni N, Seymour JR, Labbate M. Characterisation of the Pacific Oyster microbiome during a summer mortality event. Microb Ecol. 2019;77: 502–512. 10.1007/s00248-018-1226-9 29987529

[42] Colston RJ, Jackson CR. Microbiome evolution along divergent branches of the vertebrate tree of life: what is known and unknown. Mol Ecol. 2016;25: 3776–3800. 10.1111/mec.13730 27297628

[43] König S, Gros O, Heiden SE, Hinzke T, Thürmer A, Poehlein A, et al Nitrogen fixation in a chemoautotrophic lucinid symbiosis. Nat Microbiol. 2016;2: 16193 10.1038/nmicrobiol.2016.193 27775698

[44] Moulton OM, Altabet MA, Beman JM, Deegan LA, Lloret J, Lyons MK. et al Microbial associations with macrobiota in coastal ecosystems: patterns and implications for nitrogen cycling. Front Ecol Environ. 2016;14: 200–208.

[45] Belova SE, Kulichevskaya IS, Bodelier PL, and Dedysh SN. *Methylocystis bryophila* sp. nov., a facultatively methanotrophic bacterium from acidic Sphagnum peat, and emended description of the genus *Methylocystis* (ex Whittenbury et al. 1970) Bowman et al. 1993. International journal of systematic and evolutionary microbiology, 2013;63, 1096–1104. 10.1099/ijs.0.043505-0 22707532

[46] Dam B, Dam S, Blom J, and Liesack W. Genome analysis coupled with physiological studies reveals a diverse nitrogen metabolism in *Methylocystis* sp. strain SC2. PLoS One. 2013;8, e74767 10.1371/journal.pone.0074767 24130670 PMC3794950

[47] Black EM, Chimenti MS, Just CL. Effect of freshwater mussels on the vertical distribution of anaerobic ammonia oxidizers and other nitrogen-transforming microorganisms in upper Mississippi river sediment. PeerJ 2017;5: e3536 10.7717/peerj.3536 28717594 PMC5510576

[48] Augspurger T, Keller AE, Black MC, Cope WG, Dwyer FJ. Water quality guidance for protection of freshwater mussels (Unionidae) from ammonia exposure. Environ Toxicol Chem. 2003;22: 2569–2575. 10.1897/02-339 14587894

[49] Allen DC, Vaughn CC. Burrowing behavior of freshwater mussels in experimentally manipulated communities. J North Am Benthol Soc. 2009;28: 93–100.

[50] Pfeiffer JM, Atkinson CL, Sharpe AE, Capps KA, Emery KF, Page LM. Phylogeny of Mesoamerican freshwater mussels and a revised tribe‐level classification of the Ambleminae. Zool Scr. 2019;48: 106–117.

[51] Caporaso JG, Lauber CL, Costello EK, Berg-Lyons D, Gonzalez A, Stombaugh J, et al Moving pictures of the human microbiome. Genome Biol. 2011;12: R50 10.1186/gb-2011-12-5-r50 21624126 PMC3271711

